# Elicitation of Stilbenes and Benzofuran Derivatives in Hairy Root Cultures of White Mulberry (*Morus alba*)

**DOI:** 10.3390/plants12010175

**Published:** 2022-12-31

**Authors:** Lingling Fang, Amit Raj Sharma, Chineche Aniemena, Krystian Roedel, Florence Henry, Philippe Moussou, Anita Samuga, Fabricio Medina-Bolivar

**Affiliations:** 1 Arkansas Biosciences Institute, Arkansas State University, Jonesboro, AR 72401, USA; 2 Department of Biological Sciences, Arkansas State University, Jonesboro, AR 72401, USA; 3 BASF Beauty Care Solutions SAS, 69007 Lyon, France; 4 BASF Corporation, Research Triangle Park, Durham, NC 27709, USA

**Keywords:** white mulberry, stilbenes, moracins, benzofurans, prenylated stilbenes, hairy root cultures, elicitation, cyclodextrin, methyl jasmonate, hydrogen peroxide

## Abstract

Stilbene and benzofuran derivatives isolated from the root of white mulberry (*Morus alba*) have shown various biological activities, including anti-inflammatory, antioxidant, and antimicrobial properties. The objectives of this study were to develop hairy root cultures and assess the effect of multiple elicitors combinations including (I) methyl-β-cyclodextrin (CD), MgCl_2_, methyl jasmonate (MeJA), and H_2_O_2_, (II) CD, MgCl_2_, and MeJA and (III) CD, MgCl_2,_ and H_2_O_2_, on the production of these bioactive compounds. The highest yields of stilbenes and benzofurans were obtained upon co-treatment with 18 g/L CD, 3 mM H_2_O_2_ and 1 mM MgCl_2_. The stilbenes oxyresveratrol, resveratrol, and 3′-prenylresveratrol accumulated up to 6.27, 0.61, and 5.00 mg/g DW root, respectively. Meanwhile, the aryl benzofurans moracin M and moracin C accumulated up to 7.82 and 1.82 mg/g DW root, respectively. These stilbenes and benzofurans accumulated in the culture medium of the elicited hairy root cultures. They were not detected in the root tissue. However, the oxyresveratrol diglucoside mulberroside A was only detected in the root tissue with yields up to 10.01 mg/g DW. The results demonstrated that co-treatment of white mulberry hairy root cultures with multiple elicitors can significantly enhance production and secretion of stilbenes and benzofurans in this controlled and sustainable axenic culture system.

## 1. Introduction

White mulberry (*Morus alba*) is a deciduous tree native to China and widely disseminated throughout Asia, Africa, Europe, and South and North America [1,2]. Leaves, root bark, stem, and fruits of *M. alba* have been used in traditional Chinese medicine for the treatment of metabolic disorders such as diabetes, hyperlipidemia, and high blood pressure [3,4,5,6]. The chemical investigation of different tissues from white mulberry revealed that phenolic compounds, such as flavonoids, stilbenes, benzofurans, and Diels-Alder type adducts are the main constituents [7].

**Figure 1:**
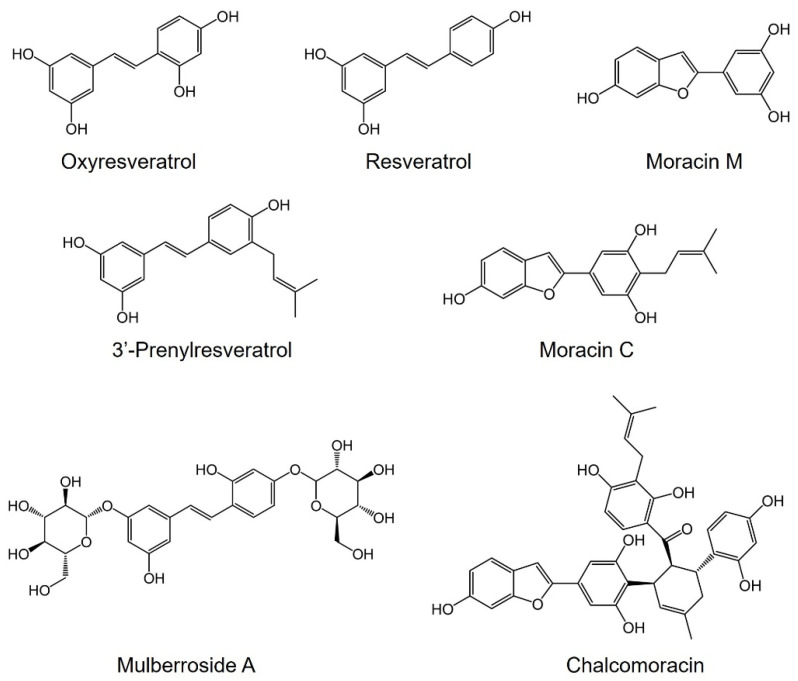
Chemical structures of stilbenes and benzofurans identified in hairy root cultures of white mulberry.

Stilbenes are polyphenolic phytoalexins produced by certain plants in response to biotic or abiotic stress. These compounds have attracted considerable attention owing to their remarkable biological properties [8]. Among these, resveratrol (*trans*-3,4′,5-trihydroxystilbene; Figure 1), has received much attention due to its simple chemical structure and potentially promising therapeutic application in various diseases [9]. Oxyresveratrol (*trans*-2,4,3′,5′-tetrahydroxy stilbene; Figure 1), another natural hydroxystilbene, exhibits more powerful antioxidant activity when compared to resveratrol due to the presence of an additional hydroxyl group [10]. Furthermore, it has shown anti-inflammatory [11,12], antiviral [13], and neuroprotective [14] activities. Oxyresveratrol, along with its diglucoside mulberroside A (Figure 1), has shown anti-browning [15], and tyrosinase inhibition [16,17,18], and therefore has been used as raw materials for skin-whitening cosmeceuticals [19].

Among the benzofurans, moracin M was originally isolated from the root bark of *M. alba*. and previous studies have described its anti-inflammatory activity [20,21]. Moracin C is one of the well-known natural 2-arylbenzofuran prenylated derivatives isolated from fungus-infected mulberry leaves [22]. It has been also identified in *Artocarpus heterophyllus* (commonly known as the jackfruit tree) [23]. Moracin C has been reported to have antibacterial [24], anticancer [25,26], antioxidant [27], α-glucosidase [28], and lipoxygenase inhibitory activities [23].

Chalcomoracin is a Diel-Alder type adduct produced by fungus-infected mulberry leaves [29]. It exhibits promising biological activities against methicillin-resistant *Staphylococcus aureus* (MRSA) [30,31], and human cancer cell lines [32,33]. It is formed through the Diels-Alder type cycloaddition of a prenylchalcone and a prenylated 2-arylbenzofuran [29]. 

The chemical composition of the mulberry tree is strongly influenced by environmental factors (such as soil and temperature) during the lengthy growth period and harvest season [34,35]. Consequently, extracts from different white mulberry accessions may exhibit non-reproducible profiles of bioactive compounds, which is a challenge for the commercial applications of this plant species. Furthermore, the variability in the chemical composition could be the reason for the limited information on the biosynthesis of specialized metabolites such as the moracins, which were originally described in mulberry. For instance, genes related to the prenylation of moracins have not been described, while flavonoid- and stilbenoid-specific prenyltransferase genes have been reported [36,37]. Therefore, there is a need to develop a sustainable bioproduction platform for white mulberry, which could provide reproducible chemical profiles for commercial applications and elucidation of the biosynthetic pathway of its bioactive compounds. 

Hairy root cultures produced via *Agrobacterium rhizogenes*-mediated transformation have emerged as a sustainable platform for the production of valuable specialized metabolites under controlled conditions [38,39]. Chemical elicitation of hairy roots is commonly used to increase the yield of metabolites. Previously, the combination of different elicitor combinations including methyl jasmonate (MeJA), methyl-β-cyclodextrin (CD), hydrogen peroxide (H_2_O_2_), and supplementation with magnesium chloride (MgCl_2_) led to the elicitation and secretion of high levels of the prenylated stilbenes arachidin-1 and arachidin-3 into the medium of hairy root cultures of peanut [40], though these multiple-elicitor treatments have not been studied in hairy root cultures of white mulberry.

In this paper, we report the establishment of hairy root cultures of white mulberry (*M. alba*) and describe three strategies to elicit the production of stilbenes and aryl benzofurans, including their prenylated derivatives. The elicitation strategies included (I) CD, MgCl_2_, MeJA, and H_2_O_2_ (II) CD, MgCl_2,_ and MeJA, and (III) CD, MgCl_2_, and H_2_O_2_. Time course studies revealed that different combinations of elicitors have a distinct effect on the levels of selected stilbenes and aryl benzofurans. 

## 2. Results

### 2.1. Development and Characterization of Hairy Root Cultures of M. alba

**Figure 2:**
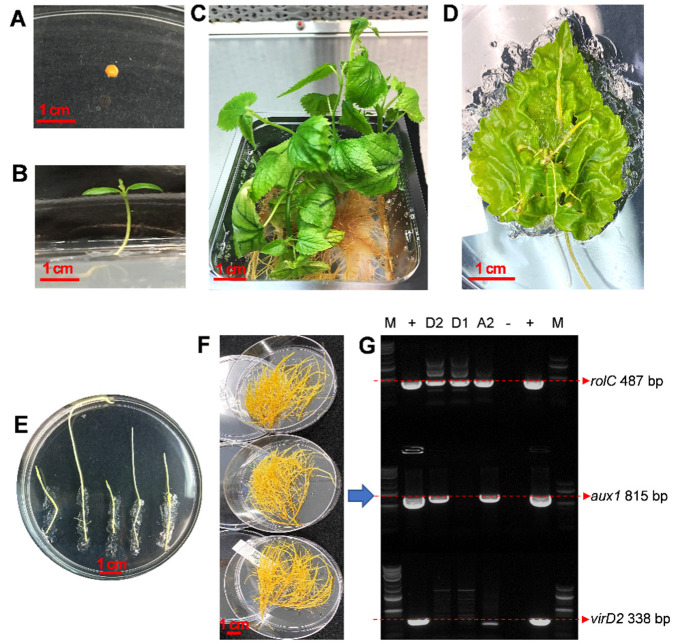
Establishment of hairy root cultures of white mulberry (*M. alba*) hairy root cultures. (**A**) Seed of white mulberry. (**B**) Two-week-old seedling. (**C**) Eight-week-old seedling. (**D**) Hairy root development from leaf infected with *Agrobacterium rhizogenes*. (**E**) Growth of hairy roots on semi-solid medium and selection based on growth. (**F**) Three selected lines after growth in liquid medium for 25 days. (**G**) PCR analysis of selected hairy root lines. Genomic DNA was isolated from hairy root lines U-A2, U-D1 and U-D2. Analyses were performed with primers targeting the *rolC, aux1* and *virD2* genes. Plasmid pRi15834 DNA was used as a positive control. ddH2O was used as a negative control. The bar in each panel corresponds to 1 cm.

Surface-sterilized seeds of white mulberry were germinated in vitro to provide aseptic plant materials for hairy root induction. The leaves of 8-week-old seedlings were excised and wounded with *A. rhizogenes* ATCC 15834 (Figure 2). After 4 to 6 weeks of inoculation, roots that developed from wounded leaves were transferred to cefotaxime-containing media to avoid agrobacteria overgrowth. After the elimination of bacteria from the hairy roots by subcultures on cefotaxime-containing media, three hairy root lines (U-A2, U-D1 and U-D2) were selected for further study due to their fast growth. The hairy roots were transferred to liquid cultures and their DNA was extracted for PCR analysis. Since *A. rhizogenes* ATCC 15834 contains two T-DNAs, T_L_-DNA (harboring *rol* genes) and T_R_-DNA (harboring *aux1* and *aux2* genes), the integration of the T_L_-DNA and T_R_-DNA into the host genome of the putative hairy roots was tested by screening for *rolC* and *aux1* genes. The PCR analysis revealed the integration of the *rolC* gene in all transformed hairy root lines. The *aux1* gene was detected in the U-D2 and U-A2 lines, but not the U-D1 line. Expression of the *rol* genes localized in T_L_-DNA is necessary for the hairy root phenotype [41]. Consequently, transfer of only T_L_-DNA is frequent in hairy roots induced by agropine strains of *A. rhizogenes* such as ATCC 15834 [42]. Similarly, in our previous study with *Arachis ipaensis* and *A. duranensis* hairy roots, some lines only included the *rol* genes from T_L_-DNA [40]. The lack of amplification of the *virD2* gene (localized outside the T-DNAs of the root-inducing plasmid) in the white mulberry hairy root lines confirmed the absence of any remaining *Agrobacterium* in the root tissue (Figure 2G). Among the three established lines, hairy root line U-D2 harboring both *rol* and *aux* genes was selected for further analysis due to its vigor and sustained growth in liquid culture.

### 2.2. Elicitation of Stilbenes and Moracins in Hairy Root Cultures of M. alba

Elicitation is a well-known strategy to induce and enhance the secretion of specialized metabolites in hairy root cultures. Our group optimized an elicitation procedure in peanut hairy root cultures to enhance the production of prenylated stilbenes. Under co-treatment with CD, MeJA, H_2_O_2,_ and MgCl_2_, the overall yield of prenylated stilbenoids in the medium reached approximately 750 mg/L [40]. 

Root and stem barks of white mulberry have been shown to accumulate prenylated stilbenes and 2-arylbenzofuran derivatives including prenylresveratrol, moracin M, and moracin C [43,44,45]. To establish a sustainable system to produce these bioactive compounds, 35-day-old hairy root cultures of white mulberry were treated with three groups of elicitors: CD + MgCl_2_ + MeJA + H_2_O_2_, CD + MgCl_2_ + MeJA and CD + MgCl_2_ + H_2_O_2_. Seven major compounds, including the stilbenes oxyresveratrol, resveratrol, 3′-prenylresveratrol and mulberroside A and arylbenzofurans and derivatives including moracin M, moracin C and chalcomoracin were detected. Identification was based on comparisons to the HPLC retention time, UV spectrum, and tandem mass spectrometry analyses of authentic standards. Since no standards were available for 3′-prenylresveratrol and chalcomoracin, their identification was based on published UV-spectra and fragmentation patterns of the mass spectrometry analyses described below. 

#### 2.2.1. Phenotype of Hairy Root Line U-D2 upon Multiple Elicitors Treatments

**Figure 3:**
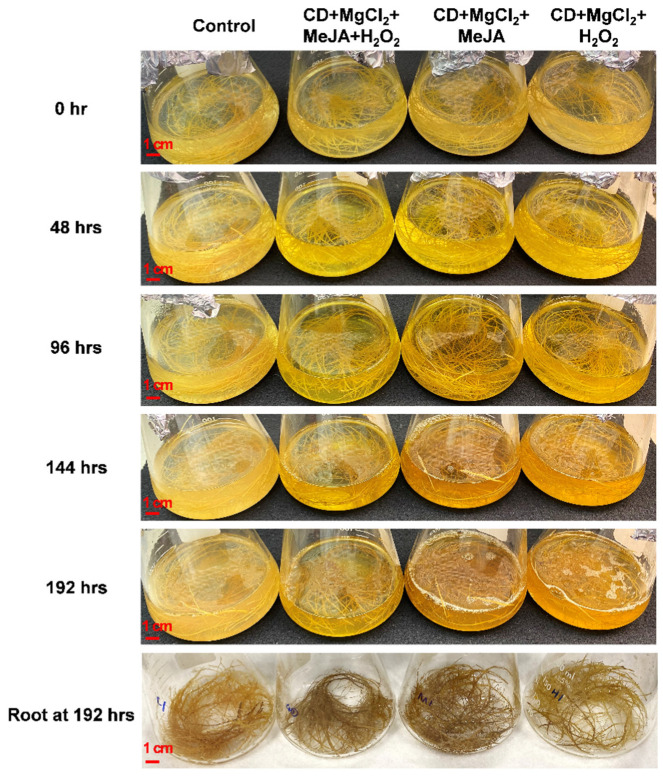
Phenotype of hairy root line U-D2 upon treatment with elicitors for different time periods. The bar in each panel corresponds to 1 cm.

The effects of the three treatments on the production of bioactive compounds were evaluated along a time course from 48 to 192 h. In contrast to the healthy root tissue observed in the non-treated control group, the hairy root tissue showed a brownish color after elicitor treatment with CD + MgCl_2_ + H_2_O_2_. Interestingly, the cultures treated with CD + MgCl_2_ + MeJA and CD + MgCl_2_ + MeJA + H_2_O_2_ showed the darkest color (Figure 3). Upon elicitation, the color of the culture medium turned to bright light-yellow color suggesting the presence of phenolic compounds in the culture medium. 

#### 2.2.2. Effect of Different Elicitor Treatments on the Yield of Stilbenes and Benzofurans

**Figure 4:**
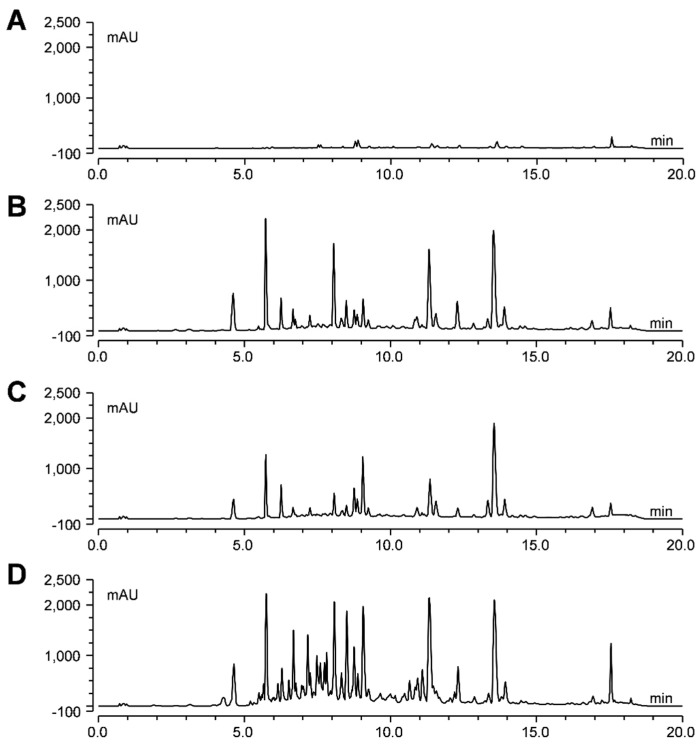
HPLC chromatograms of extracts from the medium of white mulberry line U−D2 upon different elicitation treatments at 192 h. (**A**) Non-treated control. (**B**) CD + MgCl_2_ + MeJA + H_2_O_2._ (**C**) CD + MgCl_2_ + MeJA. (**D**) CD + MgCl_2_ + H_2_O_2_. All chromatograms were monitored at 320 nm.

**Figure 5:**
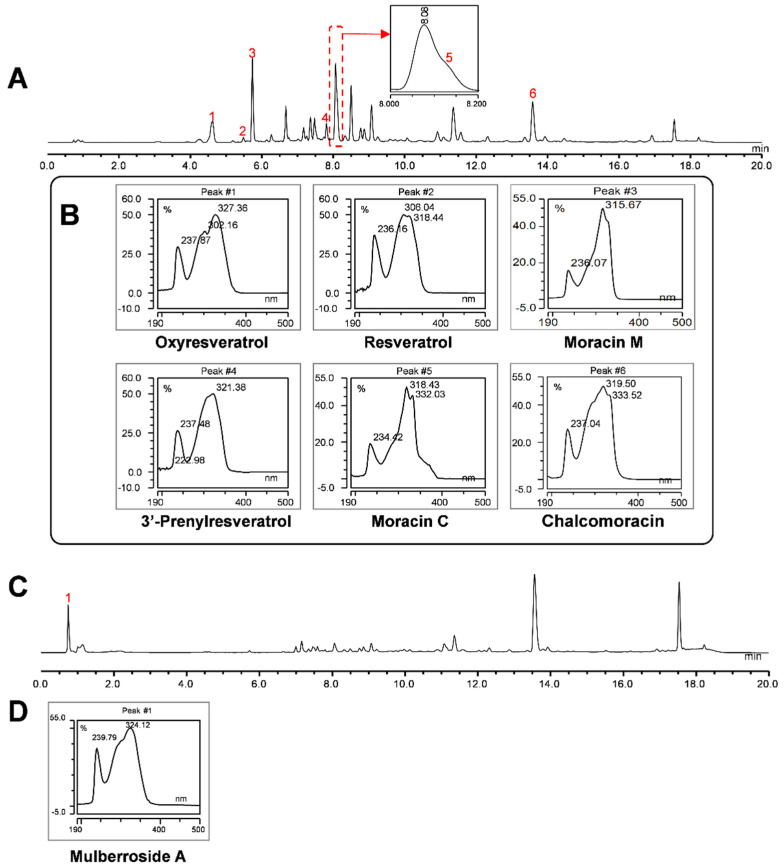
UV−spectra of compounds identified in white mulberry hairy root cultures. (**A**) HPLC chromatogram of hairy root line U−D2 medium extract after 48 h of elicitation with CD + MgCl_2_ + H_2_O_2_ (320 nm). Box shows close-up of peak from Rt 8−8.2 min. (**B**) UV−spectrum of the compounds identified in extracts of the culture medium (peaks 1−6 correspond to the red numbers shown in (**A**)). (**C**) HPLC chromatogram of hairy root line U−D2 tissue extract after 48 h of elicitation with CD + MgCl_2_ + H_2_O_2_ (320 nm). (**D**) UV-spectrum of the identified compound in the root extract.

To determine the profile of phenolic compounds, white mulberry hairy root cultures of line U-D2 were elicited with CD + MgCl_2_ + MeJA + H_2_O_2_, CD + MgCl_2_ + MeJA or CD + MgCl_2_ + H_2_O_2_, and ethyl acetate extracts of the culture medium after 48 to 192 h of elicitation were analyzed by HPLC (Figure 4 and Supplementary Figure S1). Significant amounts of phenolic compounds were found in the culture medium of the elicited hairy root cultures, whereas only very few compounds were detected in the non-treated control cultures (Figure 4). More than half of the peaks detected by HPLC were classified as stilbenes or furans based on their specific UV spectra with an absorption maximum at 320~330 nm and 315~319 nm, respectively (Figure 5). By comparing with the elution time, UV, and MS/MS spectrum of authentic standards, the compounds oxyresveratrol, resveratrol, moracin M, and moracin C were identified and quantified in the extracts from the culture medium (Figure 5).

**Figure 6:**
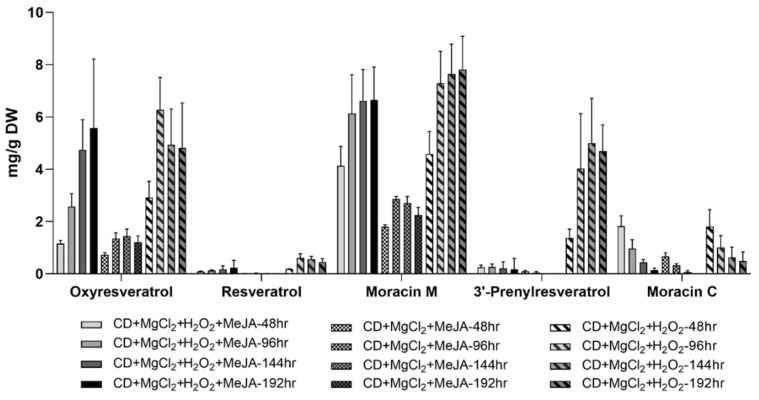
Time course of the yield of stilbenes and aryl benzofurans in the medium of white mulberry hairy root culture line U-D2 after treatment with different elicitors. Yields are expressed in mg/g DW of root tissue. Values are the average of four biological replicates and error bars represent standard deviation.

The highest yield of oxyresveratrol was 6.27 ± 1.24 mg/g DW (equivalent to 8.67 mg/L of medium) and it was found in the medium of cultures elicited with CD + MgCl_2_ + H_2_O_2_ for 96 h (Figure 6). This yield was 13.93-fold higher than that 0.45 ± 0.01 mg/g DW reported by Jeon and Choi in white mulberry leaves [43]. Since oxyresveratrol and piceatannol have equal mass, the identity of oxyresveratrol was confirmed by HPLC retention time and UV spectra analyses in addition to mass spectrometry (Supplementary Figures S5 and S6). As shown in Supplementary Figure S5, oxyresveratrol and piceatannol exhibit very different UV max. The UV max for piceatannol was 324 nm, whereas the UV max for oxyresveratrol was 327 nm. The UV max of oxyresveratrol matched the UV max of the compound detected in the extracts of the hairy root cultures of mulberry in our study (Figure 5B). Analysis of this HPLC peak designated as oxyresveratrol revealed that all parts of the peak had the UV spectrum of oxyresveratrol with a UV max of 327 nm. Furthermore, under the HPLC conditions used in this study, the piceatannol standard eluted earlier than the oxyresveratrol standard (Supplementary Figure S5). There was only one peak at the retention time corresponding to the mass of oxyresveratrol, and no nearby peaks were detected, indicating that piceatannol was not produced in these hairy root cultures. Therefore, the combination of HPLC retention time, UV spectra, and mass spectrometry analysis (Supplementary Figure S6) confirmed the presence of oxyresveratrol in the extracts from the hairy root cultures of white mulberry. Upon the same elicitation treatment with CD + MgCl_2_ + H_2_O_2_, resveratrol reached its highest yield of 0.61 ± 0.16 mg/g DW (equivalent to 0.83 mg/L of medium) in the culture medium after 96 h treatment (Figure 6). 

**Figure 7:**
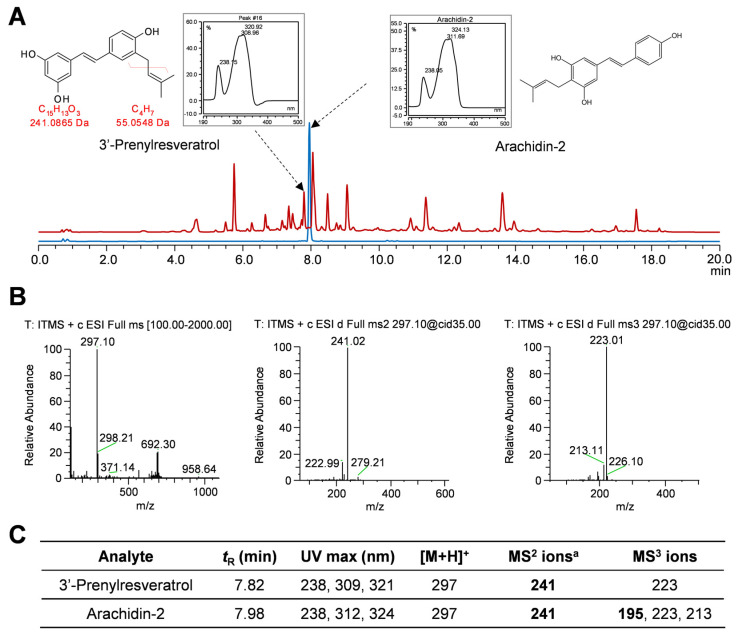
Characterization of 3′−prenylresveratrol. (**A**) HPLC chromatograms (UV 320 nm) of ethyl acetate extraction of U−D2 elicited medium (red line) and arachidin−2 standard (blue line). The UV−spectra and chemical structures of 3′−prenylresveratrol and arachidin−2 standard are shown. (**B**) HPLC−PDA−ESI−MS^3^ analysis of 3′−prenylresveratrol. (**C**) HPLC−PDA−ESI−MS^3^ fragmentation pattern of 3′−prenylresveratrol and arachidin−2. a, MS^2^ ions in boldface were the most abundant ions and were subjected to MS^3^ fragmentation.

Besides oxyresveratrol and resveratrol, another stilbene found in elicited culture medium was identified as a prenylated resveratrol with a molecular mass of 296 *m/z* (or [M + 1]^+^ of 297) (Figure 7). This compound had a distinct elution time and MS/MS fragmentation pattern when compared to arachidin-2, where the prenyl group is at the C-4 position of resveratrol, suggesting that the prenyl group might be attached to the other aromatic ring of resveratrol. To characterize this stilbene, extracts from the culture medium of the elicited hairy root cultures were run using a different HPLC column with a protocol specific for separating prenylated stilbenes (Figure 7 and Supplementary Figure S2). We confirmed that this prenylated resveratrol had same elution time, UV and MS/MS spectrum as 3-methyl-2-butenyl-3′-resveratrol (3′-prenylresveratrol) which was the product of an enzymatic reaction of a resveratrol prenyltransferase characterized from peanut [46]. The mass spectrometry analysis of this stilbene showed a molecular ion at *m/z* 297 [M + H] ^+^ and fragment ion at *m/z* 241.02 in MS^2^, confirming the presence of a prenyl moiety. The highest yield of this 3′-prenylresveratrol was 5.00 ± 1.71 mg/g DW (equivalent to 6.51 mg/L of culture medium) after 144 h treatment of CD + MgCl_2_ + H_2_O_2_ in the culture medium (Figure 6).

The aryl benzofurans moracin M and its prenylated derivative moracin C were identified and quantified in the elicited hairy root cultures of white mulberry. Moracin M accumulated to 7.82 ± 1.26 mg/g DW (equivalent to 10.33 mg/L of culture medium) after 192 h of elicitation with CD + MgCl_2_ + H_2_O_2_, while the yield of moracin C decreased from its highest yield of 1.82 ± 0.65 mg/g DW (equivalent to 2.37 ± 0.59 mg/L of culture medium) at 48 h suggesting that it might be a metabolic intermediate for other furans during the elicitation periods (Figure 6). 

**Figure 8:**
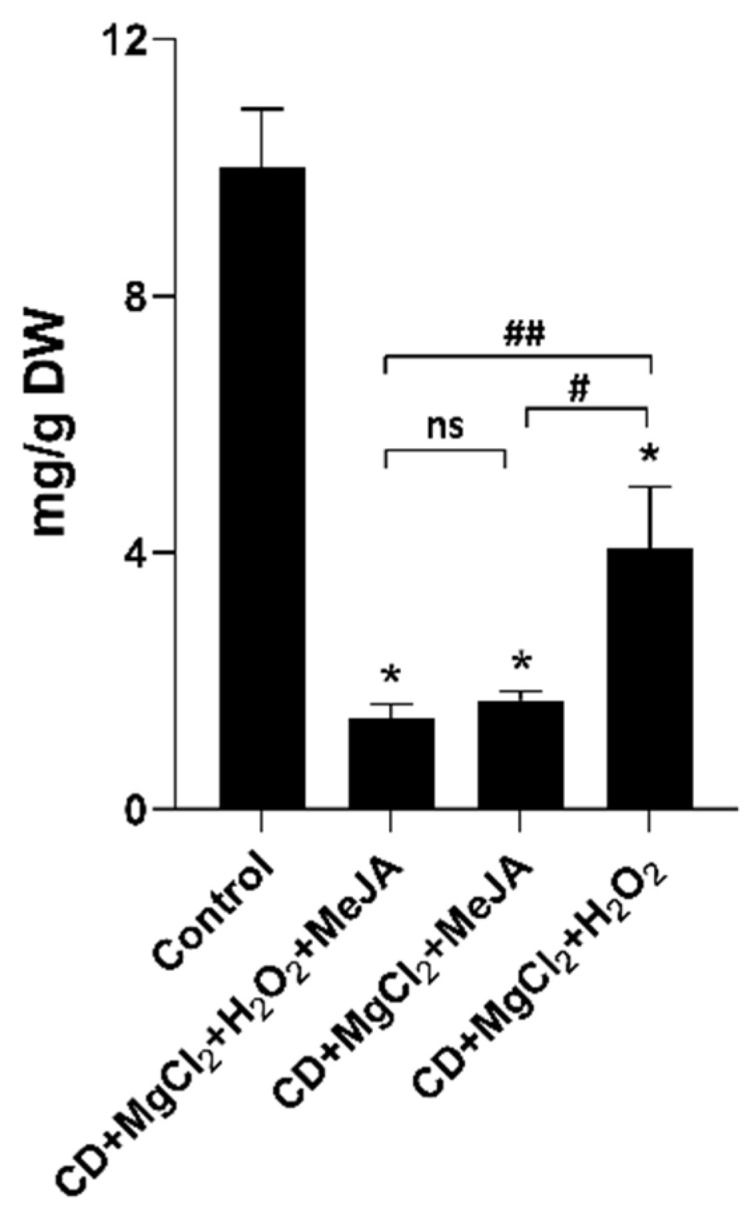
Yield of mulberroside A in the hairy root tissue after 192 h of treatment with multiple elicitors. Values are the mean of four biological replicates, and error bars represent the standard deviation. * *p* < 0.0001 treatment vs. control; # *p* < 0.001, ## *p* < 0.0005, ns, not significant among treatments.

No dramatic changes were observed in the HPLC profiles of extracts from the hairy root tissue before and after elicitation. Mulberroside A was identified in the tissue by comparing it with the retention time and UV spectrum of an authentic mulberroside A standard (Figure 5C,D). The yield of mulberroside A in the non-treated control group was 10.01 g/g DW of root, which was 2.6-fold higher than the yield in the CD + MgCl_2_ + H_2_O_2_ elicited hairy roots tissues. The latter was the highest yield among the three treatments (Figure 8).

**Figure 9:**
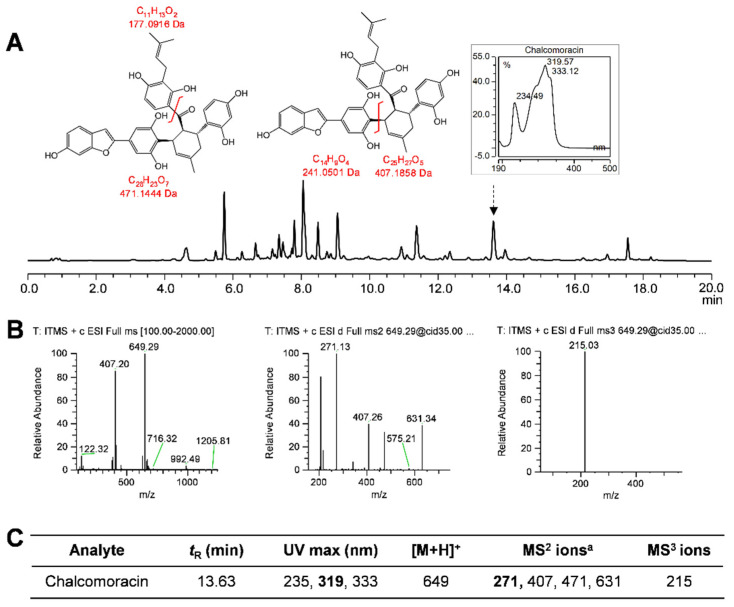
Characterization of chalcomoracin. (**A**) HPLC chromatogram (UV 320 nm) of ethyl acetate extract of the medium of white mulberry hairy root line U−D2. Chemical structure of chalcomoracin and proposed fragmentation pattern of peak identified as chalcomoracin are shown. (**B**) HPLC−PDA−ESI−MS^3^ analysis of chalcomoracin. (**C**) HPLC−PDA−MS^3^ fragmentation pattern of chalcomoracin. a, MS^2^ ions in boldface were the most abundant ions and were subjected to MS^3^ fragmentation.

Unlike the above compounds having commercial standards available, a major compound found in both the culture medium and root tissue was tentatively identified as chalcomoracin by comparing its retention time, UV-spectrum and molecular mass with those reported in the literature for chalcomoracin [43,47]. The UV spectrum of chalcomoracin exhibited an absorption maximum at 319–320 nm [43,47]. The molecular ions identified in negative ESI-MS at *m/z* 647.34 [M − H]^−^ and positive ESI-MS at *m/z* 649.29 [M + H]^+^ allowed the deduction of its molecular weight at 648 Da [43,47]. This was further corroborated by a fragmentation pattern in MS^2^ (Figure 9). 

Based on the HPLC peak area, the yield of chalcomoracin in the elicited culture medium was about 23.8-fold higher than the non-elicited culture. Whereas the yield of this compound in the elicited root tissue was 1.8-fold higher when compared to the non-elicited hairy root tissue (Supplementary Figure S3). 

Overall, when compared to the CD + MgCl_2_ + H_2_O_2_ treatment, the other two treatment groups CD + MgCl_2_ + MeJA and CD + MgCl_2_ + MeJA + H_2_O_2_ induced less accumulation of phenolic metabolites in the medium of white mulberry hairy roots. For comparison, the time course of accumulation of the identified stilbenes and aryl benzofurans expressed in mg/g DW root and mg/L of culture medium are shown in Supplementary Tables S1 and S2 and Supplementary Figure S4. The CD + MgCl_2_ + MeJA + H_2_O_2_ treatment was initially optimized in the peanut hairy root culture to enhance the yield of prenylated stilbenes. White mulberry hairy roots exhibit distinct and additional biosynthetic pathways (e.g., aryl benzofurans) when compared to peanut, which may explain the differences in response to the CD + MgCl_2_ + MeJA + H_2_O_2_ treatment.

## 3. Discussion

Chemical elicitors such as jasmonic acid, methyl jasmonate (MeJA), cyclodextrin (CD), H_2_O_2_, sodium acetate, salicylic acid, acetylsalicylic acid, ethylene, nitric oxide, sodium nitropruside, heavy metal ions, etc., have been used to induce stress in plants and consequently increase the yield and accumulation of specialized metabolites as well as produce entirely novel molecules in cell and tissue cultures [48]. A previous study with hairy root cultures has shown that the combination of different elicitors led to higher and more consistent yield of specialized metabolites when compared to a single elicitor treatment [49]. Furthermore, an orthogonal array design approach led to an optimized elicitation procedure consisting of co-treatment with 18 g/L CD, 125 µM MeJA, 3 mM H_2_O_2_ and medium supplementation with 1 mM MgCl_2_ in hairy root cultures of peanut. The latter co-treatment produced 4-fold and 2.5-fold increase in yield of the prenylated stilbenoids arachidin-1 and arachidin-3, respectively, compared to using CD alone as a elicitor [40]. Overall, enhanced production of prenylated stilbenoids was observed in peanut hairy root cultures co-treated with CD, MeJA, H_2_O_2_, and MgCl_2_ [40,50,51]. Similarly, pigeon pea hairy root cultures co-treated with CD, MeJA, H_2_O_2_, and MgCl_2_ showed high production of the bioactive prenylated flavonoid isowighteone [52]. In the current study, we established new *M. alba* hairy root cultures and treated the best growing line with three different combinations of elicitors to assess the effect on the yield of known bioactive phenolics (i.e., stilbenes and aryl benzofurans). 

Even though a few *Morus* hairy roots cultures have been reported [53,54,55], their specialized metabolite profiles have not been explored. This is the first study to report the production of stilbenes and benzofurans in hairy root cultures of white mulberry. After treatment with CD + MgCl_2_ + MeJA + H_2_O_2_, CD + MgCl_2_ + MeJA or CD + MgCl_2_ + H_2_O_2_, the stilbenes oxyresveratrol, resveratrol, 3′-prenylresveratrol, and mulberroside A and the benzofurans moracin M and moracin C were quantified. Furthermore, the relative yield change before and after treatment was also determined for chalcomoracin. Interestingly, oxyresveratrol, resveratrol, 3′-prenylresveratrol, moracin M and moracin C were only detected in the culture medium of the elicitor-treated cultures and the highest yields were obtained upon treatment with CD + MgCl_2_ + H_2_O_2_. Under this treatment, most of the identified stilbenes and benzofurans reached their highest yield after 192 h. Interestingly, mulberroside A levels were reduced upon all elicitor treatments. The latter is a glucoside of oxyresveratrol that accumulated in the tissue of the hairy roots and was not detected in the culture medium. Elicitation may activate the hydrolysis of this glucoside to release oxyresveratrol, and consequently leading to its secretion and increased accumulation in the culture medium. In addition, elicitation may also induce de novo synthesis of oxyresveratrol. Both factors may contribute to the production of oxyresveratrol and additional studies are needed to elucidate this mechanism. 

To enhance the production of specialized metabolites in *Morus* species, different treatments such as MeJA, yeast extract, chitosan, salicylic acid and UV have been assessed by different groups. *M. alba* root cultures were co-elicited with MeJA and yeast extract leading to higher production of mulberroside A (30.3 ± 2.68 mg/g DW), oxyresveratrol (68.6 ± 3.53 µg/g DW), and resveratrol (10.2 ± 0.53 µg/g DW) when compared to those treated with MeJA or yeast extract alone [56]. Notably, our current study showed a 91-fold higher amount of oxyresveratrol (6.27 ± 1.24 mg/g DW) and 60-fold higher amount of resveratrol (0.61 ± 0.16 mg/g DW) accumulation when *M. alba* hairy root cultures co-treated with CD + MgCl_2_ + H_2_O_2_ for 96 h. Importantly, when compared to the non-elicited hairy root cultures, all three elicitor treatments (i.e., CD + MgCl_2_ + MeJA + H_2_O_2_, CD + MgCl_2_ + MeJA or CD + MgCl_2_ + H_2_O_2_) led to induction and accumulation of oxyresveratrol in the culture medium and among these treatments, CD + MgCl_2_ + H_2_O_2_ produced the highest yield of oxyresveratrol. This stilbene was not detected in the non-elicited cultures. The genes and corresponding enzymes involved in the biosynthesis of oxyresveratrol have not been identified. Though, Wang et al. reported that the MaSTS3 gene isolated from mulberry (*Morus atropurpurea*) encoded for a stilbene synthase involved in the biosynthesis of resveratrol [57]. That study speculated that oxyresveratrol is produced upon oxidation or another type of derivatization of resveratrol. Additional studies are needed to identify the genes/enzymes involved in the biosynthesis of oxyresveratrol and elucidate the direct roles of the multiple elicitor treatments and their components on the expression of these genes in the white mulberry hairy roots. 

Regarding the benzofurans, Jeon and Choi reported that leaves of white mulberry treated with UV accumulated moracin M and moracin C at 0.17 ± 0.0.006 mg/g DW and 0.07 ± 0.0.003 mg/g DW, respectively [43]. Similarly to the stilbenes, the hairy root cultures of *M. alba* treated with CD + MgCl_2_ + H_2_O_2_ accumulated 46-fold and 26-fold higher levels of moracin M (7.82 ± 1.26 mg/g DW) and moracin C (1.82 ± 0.65 mg/g DW). 

Several metabolites of *Morus* species are known to have a wide spectrum of biological properties, including antibacterial, antifungal, analgesic, anti-depressant, and antioxidation properties [58,59,60]. In addition to the characterized metabolites of the current study, several other compounds were also present in the extracts of the medium of the elicited hairy root cultures. Analyses of the UV spectra of these compounds suggest that they belong to the stilbene and benzofuran family. Thus, this white mulberry hairy root bioproduction platform would be promising for mining more metabolites with potential new bioactivities.

Despite exhibiting broad biological activities, the biosynthetic pathway of moracins, particularly prenylated moracins, remains unclear. Prenylation of moracin M, the non-prenylated precursor, is a key step in the biosynthesis of prenylated moracins. Studies have shown that prenyltransferases catalyze the prenylation of various substrates, including flavonoids, stilbenoids, and other phenolic compounds with strong substrate specificity. Until now, a flavonoid-specific prenyltransferase (MaIDT) [36] and a stilbenoid-specific prenyltransferase (MaOGT) [37] have been the only characterized phenolic prenyltransferases from white mulberry. To elucidate their biosynthetic pathway, it is crucial to develop a controlled bioproduction system for the consistent production of moracins. Recently, the first stilbenoid-specific prenyltransferase genes were identified and characterized in elicitor-treated peanut hairy root cultures by our group [46,61]. This strategy could also apply for the identification of moracin-specific prenyltransferases using the white mulberry hairy root culture described in this study. 

## 4. Materials and Methods

### 4.1. Sterilization and Germination of White Mulberry Seeds

Seeds of white mulberry (*M. alba* var. tatarica; source country: Ukraine) were obtained from the Sheffied’s seed Company https://sheffields.com/ (accessed on 13 July 2019), New York, NY, USA. Dry seeds were soaked in 50 °C warm water in a 50 mL conical tube overnight. The warm water cooled down naturally. Any remaining fruit tissue outside the seeds was removed before starting the surface sterilization. Then, the seeds were dipped into 70% ethanol for 3 min, followed by 66.7% Clorox (4% *v*/*v* NaOCl) with 0.05% tween-20 for 15 min. Afterward, the seeds were put back in 70% ethanol for 1 min and rinsed thoroughly with sterilized distilled water 4–5 times. The sterilized seeds were put on plates containing MS [62] basal media with 30 g/L sucrose and 4 g/L phytagel and cultured at 24 °C under darkness until germination.

### 4.2. Establishment of Hairy Root Lines of White Mulberry

Fresh leaves were excised from in vitro seedlings of white mulberry established as described above and wounded with a scalpel containing *Agrobacterium rhizogenes* strain ATCC 15834. The wounded leaves were cultured on MSV medium [63] for 2–3 days until bacterium growth was observed around the wounded area. Then, the leaves were subcultured on MSV medium with 200 mg/L cefotaxime. Leaves were maintained in this medium until hairy roots developed. After hairy roots reached about 2 cm in length, they were excised from the leaves and placed onto a fresh MSV plate with 200 mg/L cefotaxime. Newly grown hairy root tips were cut and placed onto a MSV plate without cefotaxime. After eliminating *Agrobacterium*, the hairy roots were subcultured into flasks containing liquid MSV medium.

Among the several hairy root lines established, lines U-A2, U-D1 and U-D2 were selected for their better growth and used for PCR analyses. The hairy roots were lyophilized in a Freeze Dry System Freezone 4.5 lyophilizer (Labconco™, Kansas City, MO, USA) and then the lyophilized hairy root tissue was used to extract genomic DNA using the DNeasy^®^ Plant Mini kit (Qiagen, Germantown, MD, USA). PCR analyses of rolC, aux1, and virD2 genes were performed as described before [64].

### 4.3. Elicitation of Hairy Root Cultures

Thirty-five-day old hairy root cultures of line U-D2 grown in 250 mL flasks with 50 mL of MSV medium were used for elicitation. The old spent culture medium was removed and replaced by 100 mL fresh MSV medium containing three different groups of elicitors. Group-I: 18 g/L methyl-β-cyclodextrin (CD; CAVASOL^®^ W7 M, Wacker, Munich, Germany), 125 µM methyl jasmonate (MeJA; Sigma-Aldrich, St. Louis, MO, USA), 3 mM H_2_O_2_ (Fisher Scientific, Waltham, MA, USA), and additional 1 mM MgCl_2_ (Sigma-Aldrich, St. Louis, MO, USA), group-II: 18 g/L CD, 125 µM MeJA, and additional 1 mM MgCl_2_, and group-III: 18 g/L CD, 3 mM H_2_O_2_ and additional 1 mM MgCl_2_. A control group was also analyzed by only refreshing MSV medium without adding any of the elicitors mentioned above. Each group had four flasks as biological replicates.

### 4.4. Extraction of Phenolics from the Hairy Root Culture Medium and Tissue

A time-course elicitation experiment was conducted. Five mL aliquots of medium were collected at 48, 96, 144 and 192 h after elicitor treatment. The aliquots were mixed with 5 mL of ethyl acetate in a 15 mL conical tube by vortexing for 30 s. After centrifugation at 3000 rpm (Eppendorf Centrifuge 5810R, Eppendorf, Hamburg, Germany) for 5 min, the upper organic phase was transferred to borosilicate glass tubes (Fisherbrand^TM^, Fisher Scientific) and dried in a SpeedVac at 40 °C. The dried residue was resuspended in 500 µL of MeOH and analyzed by reversed-phase HPLC as detailed below.

To extract the phenolic compounds from the root tissue after the elicitation treatment, the hairy roots were first rinsed with water thoroughly to get rid of any remaining medium. The hairy roots were frozen at −80 °C and then lyophilized. The dried hairy roots were ground in a mortar with a pestle. Forty mg of dried root tissue was extracted with 2 mL 95 % ethanol. After centrifugation at 12,000 rpm (Thermo Scientific^TM^ Sorvall^TM^ Legend^TM^ Micro 21) for 10 min, the supernatant was analyzed by HPLC.

### 4.5. HPLC Analysis

HPLC analyses were performed in an Ultimate 3000 UHPLC system (Thermo Fisher Scientific, Waltham, MA, USA). The Waters ACQUITY UPLC HSS T3 Column (100Å, 1.8 µm, 2.1 mm × 100 mm, SKU: 186003539, Waters Corporation, Milford, MA, USA) was used for the separation of various compounds in the samples. Chromatography was done at 40 °C with a flow rate of 0.4 mL/min. A mobile phase consisting of acetonitrile (A) and water with 1% formic acid (B) was used. The column was initially equilibrated with 15% A and 85% B for 1 min. Then, a gradient was performed from 15% A and 85% B to 50% A and 50% B (1−4 min), followed by a gently linear gradient from 50% A and 50% B to 65 % A and 35 % B (4–14 min), and after a further increased to 100% A within 2 min (14–16 min), the mobile phase was returned to the initial condition for another 4 min (16–20 min). Reference compounds for establishing standard curves included mulberroside A, *trans*-resveratrol and oxyresveratrol procured from Cayman (Ann Arbor, MI, USA), whereas moracin M and moracin C were procured from 1PlusChem (San Diego, CA, USA). Arachidin-2 standard (>95% purify determined by HPLC under absorbance at 320 nm and 340 nm) was purified from elicited peanut hairy root culture medium as described before [60]. Dilutions of the standards were made in MeOH to obtain calibration curves for quantitative analysis, with the exception of mulberroside A which was dissolved in 95% ethanol. Calibration curves were established using absorbance at 320 nm for all compounds.

### 4.6. Liquid Chromatography-Mass Spectrometry Analysis

The UltiMate 3000 ultra-high-performance liquid chromatography (UHPLC) system (Thermo Scientific, Waltham, MA, USA) was used for chromatography. The chromatographic separation method followed the same HPLC conditions as described above, except 0.5% formic acid was used. The column temperature was maintained at 40 °C. The flow rate was 0.4 mL/min, and the injection volume was 10 μL. UV chromatograms were recorded at 265 and 320 nm. Mass spectrometry was performed on a LTQ XL linear ion trap mass spectrometer (Thermo Scientific, Waltham, MA, USA) with an electrospray ionization (ESI) source. Ultrahigh pure helium (He) was used as the collision gas and high purity nitrogen (N_2_) as the sheath and auxiliary gas. All mass spectra were performed in both positive and negative ion modes with ion spray voltage at 4 kV, sheath gas at 45 arbitrary units (AU), auxiliary gas at 15 AU, and capillary temperature at 300 °C. Full mass scans were recorded in the range *m/z* 100–2000. Collision-induced dissociation (CID) was used for the breakage of the molecular ion into smaller fragments. The relative collision energy was set at 35% of the maximum to produce optimum yields of fragment ions. The data were analyzed using the Xcalibur software (Thermo Scientific, Waltham, MA, USA).

## 5. Conclusions

To our knowledge, this is the first report on the substantial production of stilbene and benzofurans in chemically elicited hairy root cultures of *M. alba*. Upon treatment with CD + MgCl_2_ + MeJA + H_2_O_2_, CD + MgCl_2_ + MeJA or CD + MgCl_2_ + H_2_O_2_ the *M. alba* hairy root can produce and secrete into the culture medium high levels of resveratrol, oxyresveratrol, moracin C and moracin M. Furthermore, these compounds were not detected in the non-elicited hairy root cultures. Among the three treatments, CD + MgCl_2_ + H_2_O_2_ led to the highest yield of these specialized metabolites. Several other unknown compounds were also induced and secreted into the culture medium, holding the potential for the discovery of novel bioactive compounds. 

## Data Availability

The data of this work are available upon request.

## Supplementary Materials

The following supporting information can be downloaded at: https://www.mdpi.com/article/10.3390/plants12010175/s1, Figure S1. HPLC chromatograms of extracts from the medium and tissue of hairy root cultures of white mulberry line U-D2 after different elicitation treatments; Figure S2. HPLC analyses of the medium extract from elicited hairy root cultures of white mulberry (CD + MgCl_2_ + H_2_O_2_, 144 h) and arachidin-2 standard; Figure S3. Quantification of HPLC peak area of chalcomoracin detected in white mulberry hairy root line U-D2; Figure S4. Time course of yield of stilbenes and aryl benzofuran in the medium of white mulberry hairy root culture line U-D2 after treatment with different of elicitors; Figure S5. HPLC analyses of piceatannol and oxyresveratrol standards; Figure S6. Mass spectrometry analysis of oxyresveratrol standard (left) and oxyresveratrol identified in extract from the medium of elicited hairy root culture of white mulberry; Table S1. Yield of the secreted compounds in the culture medium of white mulberry hairy roots. The yields are in mg/g DW of root tissue; Table S2. Yield of the secreted compounds in the culture medium of white mulberry hairy roots. The yields are in mg/L of culture medium.
