# Supplementary material for: Elicitation of Stilbenes and Benzofuran Derivatives in Hairy Root Cultures of White Mulberry (Morus alba)
Source: Plants (Basel). 2022 Dec 31;12(1):175. doi: 10.3390/plants12010175 (PMC9823389; doi:10.3390/plants12010175)
Supplement: Supplementary file 1 [file plants-12-00175-s001.zip › plants-1960617-supplementary.pdf]

Supplementary Information

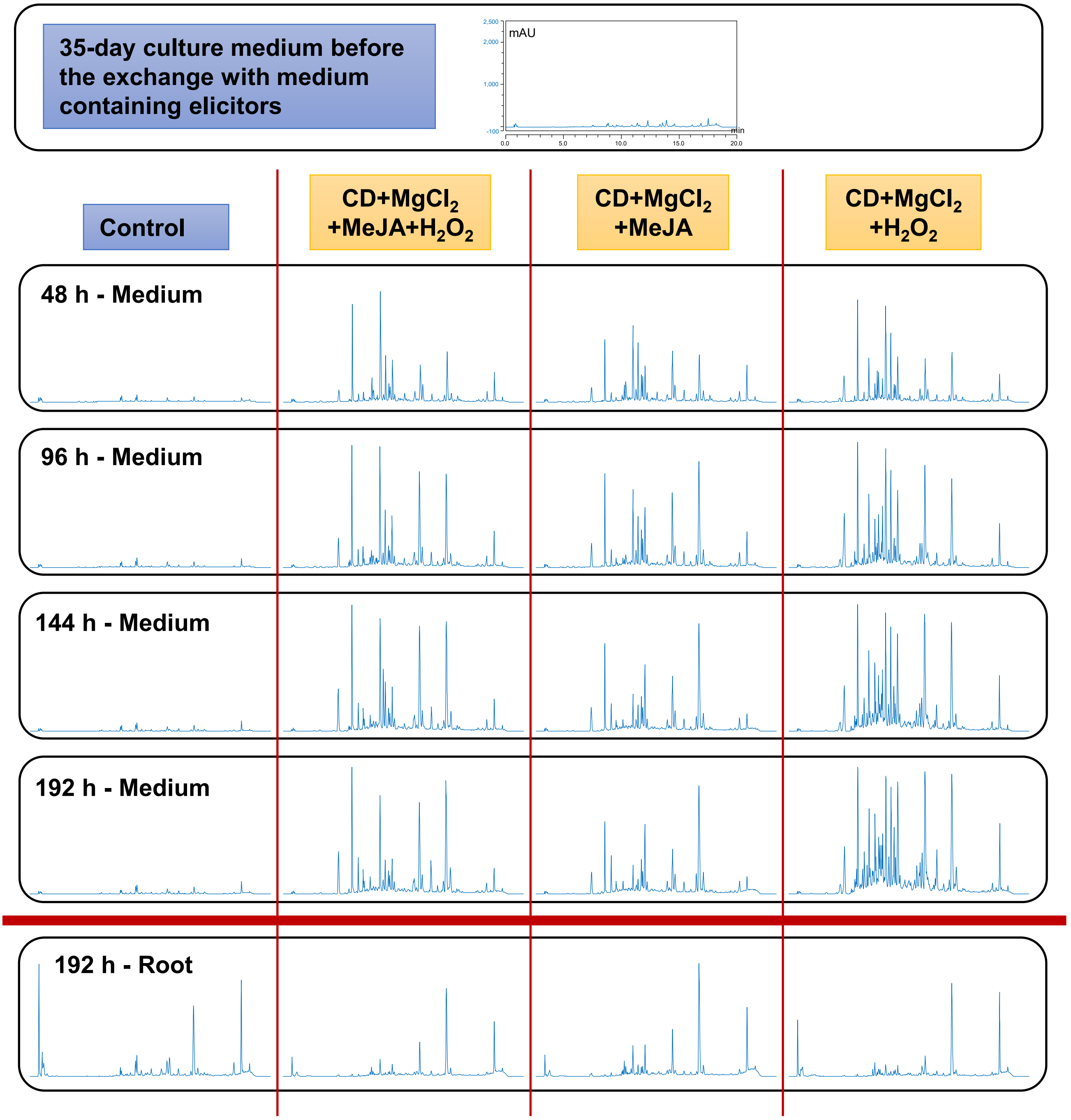

Supplementary Figure S1. HPLC chromatograms of extracts from the medium and tissue of hairy root cultures of white mulberry line U-D2 after different elicitation treatments. *Top:* Chromatogram of the medium extract before elicitation. *Control:* Chromatogram of medium extracts after medium exchange without elicitors. The extracts of the medium were concentrated 10-fold before HPLC analysis. All chromatograms were monitored at 320 nm. The y-axis of each chromatogram is at the same scale from -100 mAU to 2500 mAU. CD: methyl-β-cyclodextrin; MeJA: methyl jasmonate.

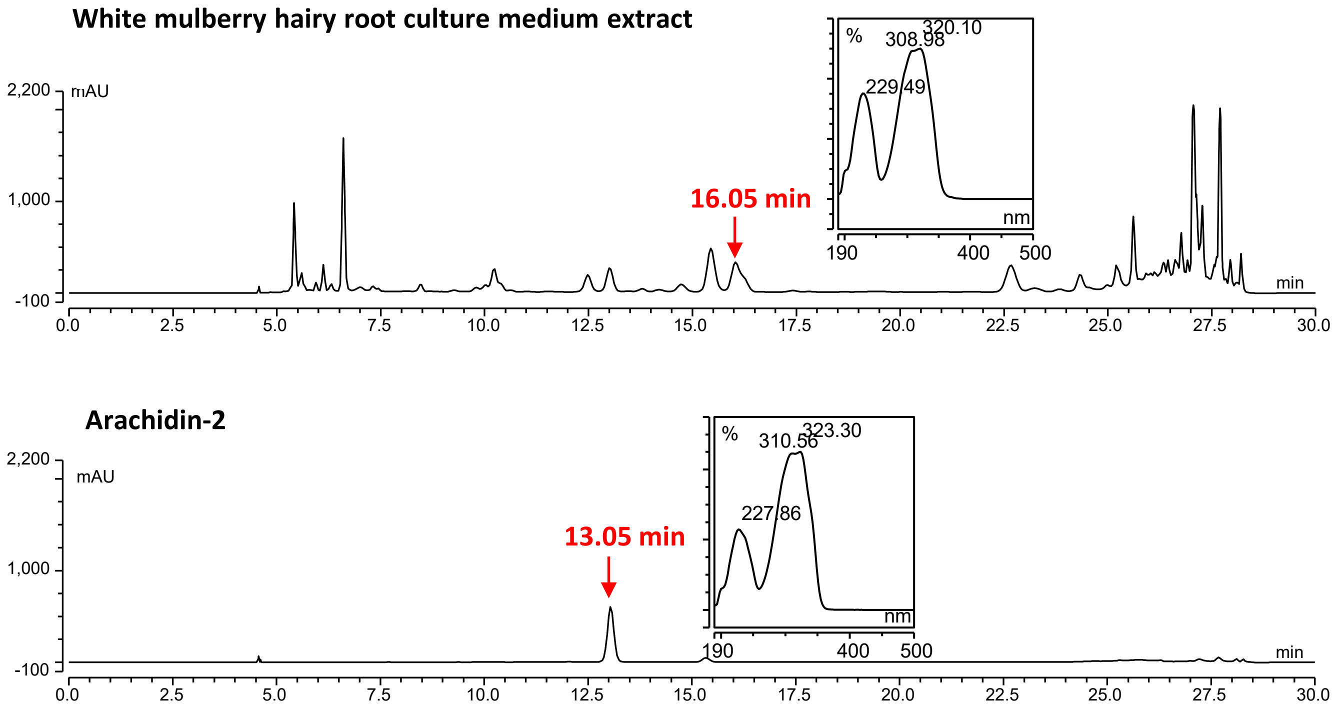

Supplementary Figure S2. HPLC analyses of the medium extract from elicited hairy root cultures of white mulberry (CD+$MgCl_2$+H_2O_2, 144 h) and arachidin-2 standard. Samples were analyzed using conditions optimized for peanut stilbenes. CD: methyl-β-cyclodextrin; MeJA: methyl jasmonate.

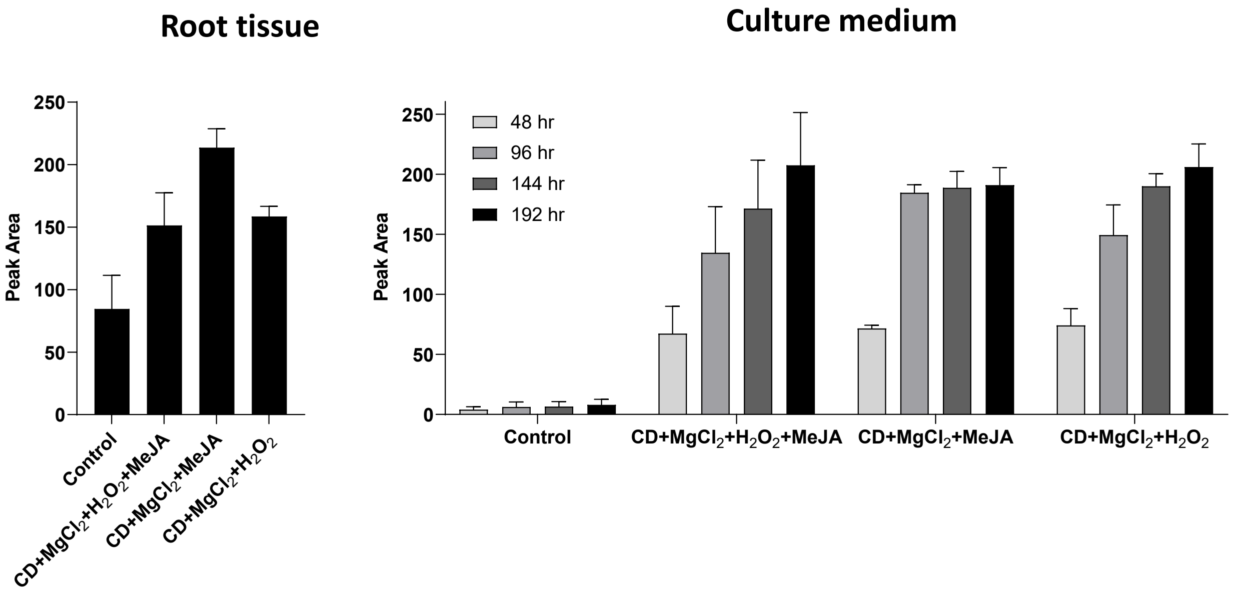

Supplementary Figure S3. Quantification of HPLC peak area of chalcomoracin detected in white mulberry hairy root line U-D2. CD: methyl-β-cyclodextrin; MeJA: methyl jasmonate.

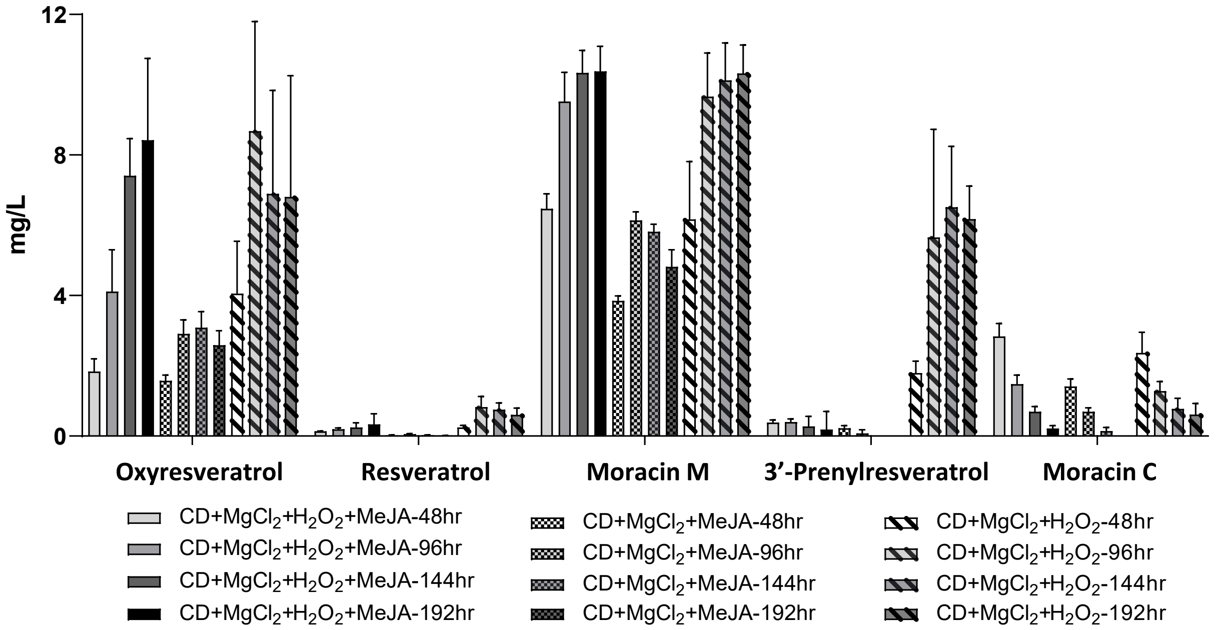

Supplementary Figure S4. Time course of yield of stilbenes and aryl benzofurans in the medium of white mulberry hairy root culture line U-D2 after treatment with different elicitors. Yields are expressed in mg/L of culture medium. Values are the average of four biological replicates and error bars represent standard deviation. CD: methyl-β-cyclodextrin; MeJA: methyl jasmonate.

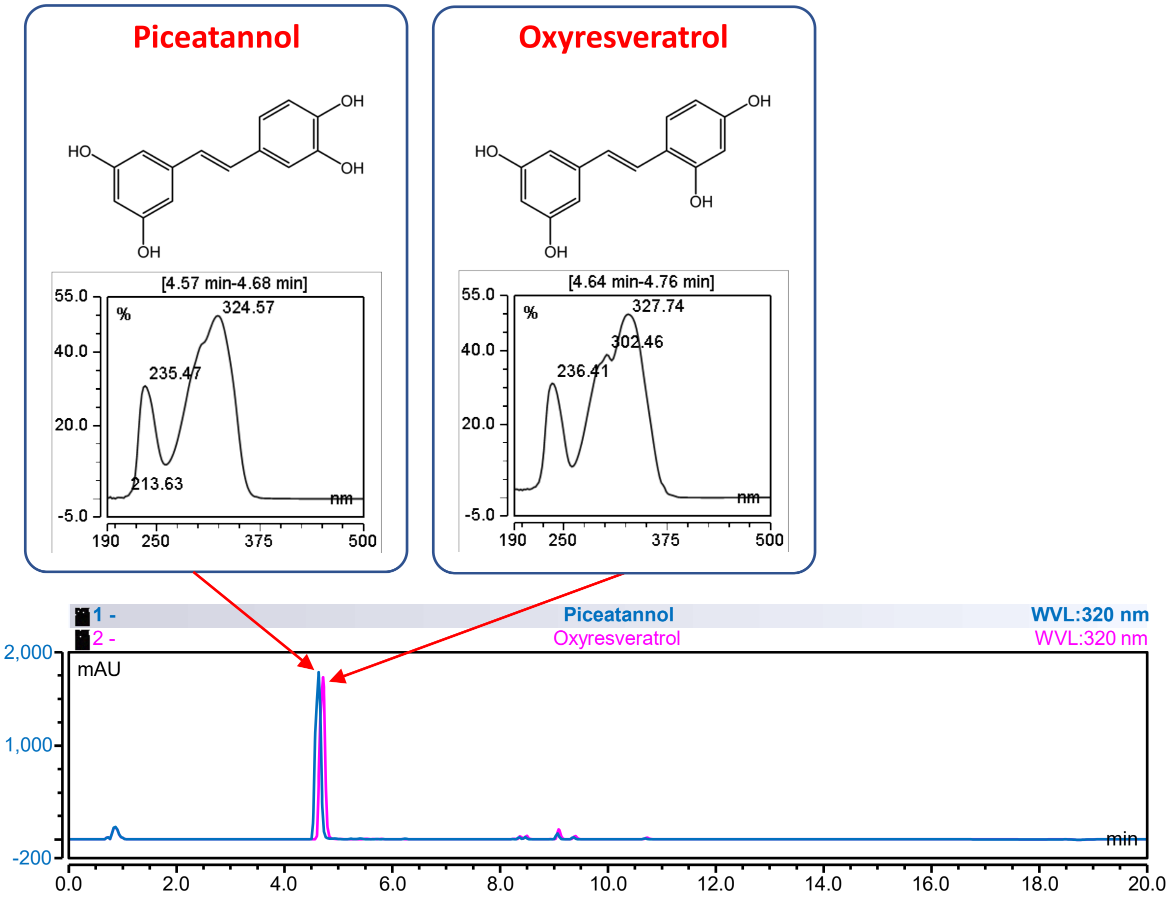

Supplementary Figure S5. HPLC analyses of piceatannol and oxyresveratrol standards.

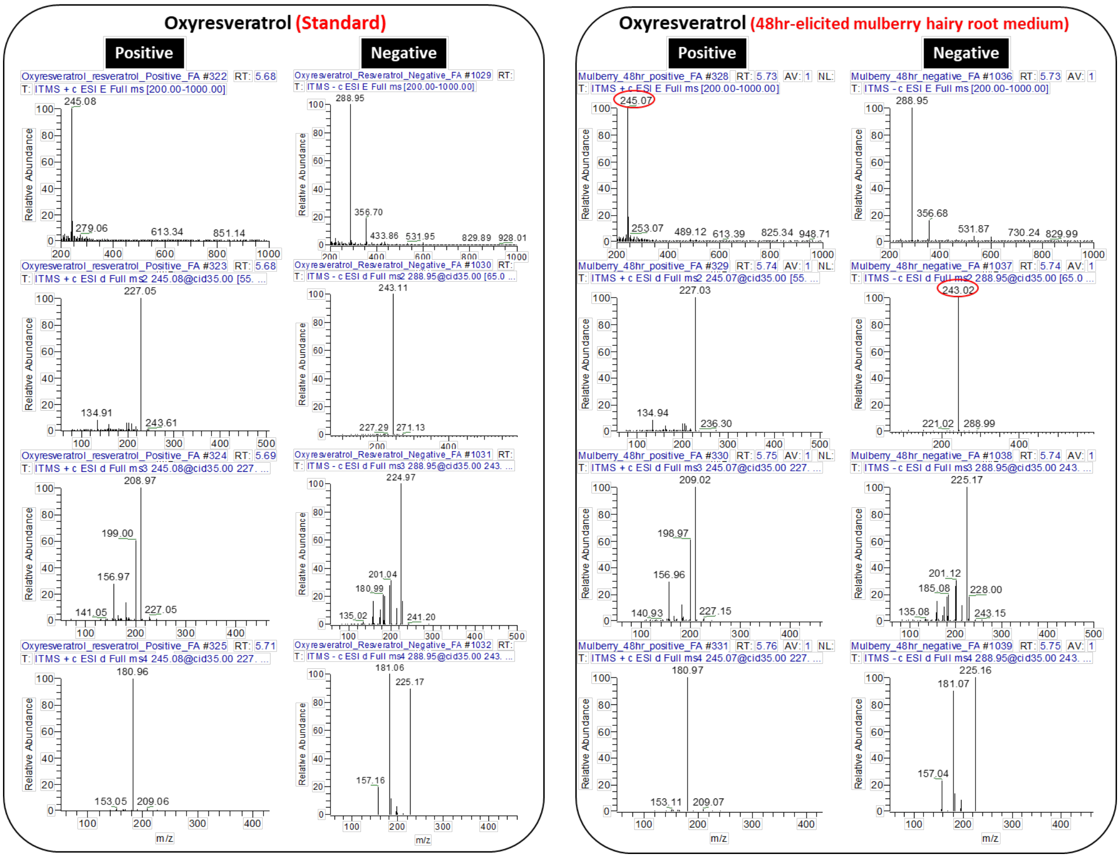

Supplementary Figure S6. Mass spectrometry analysis of oxyresveratrol standard (*left*) and oxyresveratrol identified in extract from the medium of elicited hairy root culture of white mulberry, line U-D2 (*right*). Elicitation was done with CD+$MgCl_2$+H_2O_2 for 48 hours.

Supplementary Table S1. Yield of stilbenes and benzofurans in the medium of white mulberry hairy root cultures, line U-D2. The yields are the average of four biological replicates expressed in mg/g DW of root tissue.

Secreted Compounds in U-D2 Elicited Medium (mg/g DW of root tissue)

	Oxyresveratrol	Resveratrol	Moracin M	3′-Prenylresveratrol	Moracin C
CD+$MgCl_2$+H_2O_2+MeJA-48hr	1.15±0.13	0.09±0.02	4.14±0.74	0.25±0.08	1.82±0.40
CD+$MgCl_2$+H_2O_2+MeJA-96hr	2.57±0.50	0.13±0.03	6.14±1.48	0.27±0.11	0.97±0.34
CD+$MgCl_2$+H_2O_2+MeJA-144hr	4.75±1.14	0.17±0.13	6.62±1.20	0.21±0.25	0.44±0.11
CD+$MgCl_2$+H_2O_2+MeJA-192hr	5.57±2.64	0.24±0.27	6.65±1.26	0.17±0.42	0.15±0.08
CD+$MgCl_2$+MeJA-48hr	0.73±0.08	0.01±0.00	1.79±0.08	0.10±0.03	0.66±0.14
CD+$MgCl_2$+MeJA-96hr	1.36±0.21	0.03±0.01	2.86±0.10	0.04±0.05	0.32±0.06
CD+$MgCl_2$+MeJA-144hr	1.44±0.27	0.01±0.01	2.71±0.24	-	0.07±0.05
CD+$MgCl_2$+MeJA-192hr	1.21±0.24	0.01±0.01	2.25±0.29	-	-
CD+$MgCl_2$+H_2O_2-48hr	2.93±0.62	0.18±0.01	4.59±0.86	1.37±0.35	1.82±0.65
CD+$MgCl_2$+H_2O_2-96hr	6.27±1.24	0.61±0.16	7.30±1.22	4.03±2.10	1.01±0.45
CD+$MgCl_2$+H_2O_2-144hr	4.93±1.38	0.56±0.11	7.65±1.14	5.00±1.71	0.63±0.39
CD+$MgCl_2$+H_2O_2-192hr	4.82±1.71	0.45±0.13	7.82±1.26	4.69±1.01	0.49±0.35

Supplementary Table S2. Yield of stilbenes and benzofurans in the medium of white mulberry hairy root cultures, line U-D2. The yields are the average of four biological replicates expressed in mg/L of culture medium.

Secreted Compounds in U-D2 Elicited Medium (mg/L)					
	Oxyresveratrol	Resveratrol	Moracin M	3′-Prenylresveratrol	Moracin C
CD+$MgCl_2$+H_2O_2+MeJA-48hr	1.84±0.36	0.14±0.01	6.47±0.43	0.38±0.07	2.84±0.37
CD+$MgCl_2$+H_2O_2+MeJA-96hr	4.11±1.19	0.20±0.03	9.52±0.83	0.40±0.09	1.48±0.26
CD+$MgCl_2$+H_2O_2+MeJA-144hr	7.41±1.06	0.25±0.13	10.34±0.64	0.28±0.29	0.69±0.14
CD+$MgCl_2$+H_2O_2+MeJA-192hr	8.42±2.33	0.34±0.30	10.38±0.71	0.19±0.51	0.22±0.08
CD+$MgCl_2$+MeJA-48hr	1.57±0.17	0.02±0.01	3.85±0.14	0.22±0.08	1.41±0.21
CD+$MgCl_2$+MeJA-96hr	2.91±0.39	0.05±0.02	6.14±0.24	0.08±0.10	0.69±0.11
CD+$MgCl_2$+MeJA-144hr	3.09±0.45	0.02±0.01	5.81±0.21	-	0.14±0.10
CD+$MgCl_2$+MeJA-192hr	2.59±0.41	0.01±0.01	4.82±0.49	-	-
CD+$MgCl_2$+H_2O_2-48hr	4.06±1.49	0.25±0.05	6.17±1.64	1.80±0.33	2.37±0.59
CD+$MgCl_2$+H_2O_2-96hr	8.68±3.12	0.83±0.30	9.66±1.24	5.66±3.08	1.28±0.27
CD+$MgCl_2$+H_2O_2-144hr	6.90±2.94	0.75±0.19	10.13±1.07	6.51±1.73	0.77±0.30
CD+$MgCl_2$+H_2O_2-192hr	6.81±3.45	0.61±0.19	10.33±0.80	6.18±0.94	0.61±0.31
